# Severe Phytophotodermatitis Caused by Mulberry Tree: A Case Report and Literature Review

**DOI:** 10.1055/a-2699-8042

**Published:** 2026-01-30

**Authors:** Young Geun Kim, Keun-Cheol Lee

**Affiliations:** 1 Gang Hospital, Public Health Doctor, Goseong-gun, Korea (the Republic of); 2 Department of Plastic and Reconstructive Surgery, College of Medicine, Dong-A University, Busan, South Korea

**Keywords:** phytophotodermatitis, mulberry tree, furocoumarins, phototoxic reaction

## Abstract

Phytophotodermatitis (PPD) is an uncommon dermatologic condition that occurs after exposure to furocoumarins found in certain plant saps, which become activated upon ultraviolet A radiation exposure. This condition is frequently misdiagnosed as cellulitis, allergic dermatitis, or other common skin disorders. Here, we present a case of severe PPD in a 52-year-old male who initially developed a rash and blistering on his forearm following outdoor activity. He was initially misdiagnosed with cellulitis; however, his symptoms persisted despite antibiotic treatment, prompting further evaluation. A detailed patient history revealed recent contact with a mulberry tree, and together with characteristic biopsy findings, confirmed the diagnosis of PPD. The patient showed significant improvement following the administration of oral corticosteroids and topical steroid treatment. This case underscores the importance of recognizing PPD in clinical practice to prevent misdiagnosis and ensure effective management.

## Introduction

Phytophotodermatitis (PPD), a rare dermatological condition, is often overshadowed by more common skin conditions such as contact dermatitis, allergic dermatitis, and cellulitis. 1 2 3 This condition arises from a phototoxic reaction involving compounds, primarily furocoumarins or psoralens, 4 found in the sap of certain plants. Upon exposure to ultraviolet A radiation (UVA; wavelengths 320–400 nm), these compounds become reactive, leading to distinctive clinical manifestations, typically characterized by erythema and bullae formation. 4 5 In severe cases, it can progress to eschar formation and even full-thickness skin loss. 6 7

The diagnosis of PPD poses significant challenges due to its clinical resemblance to more common dermatological conditions. 3 8 Accurate diagnosis hinges on a thorough patient history and awareness of its unique clinical features. As a result, it is frequently misdiagnosed, leading to inappropriate or delayed treatment. This paper aims to increase clinical awareness of PPD by presenting a detailed case study and reviewing the literature. Through this exploration, we seek to enhance the understanding and recognition of PPD within the field of dermatology and wound care.

## Case

A 52-year-old male with a history of diabetes presented to the emergency room with a scratch wound on his right forearm, sustained 3 days earlier while repairing utility poles outdoors. On that hot summer day, he had scraped his right forearm against trees and the pole while wearing a short-sleeved shirt and gloves. The next day, a pruritic rash developed on his right forearm, soon followed by blistering.

**Fig. 1:**
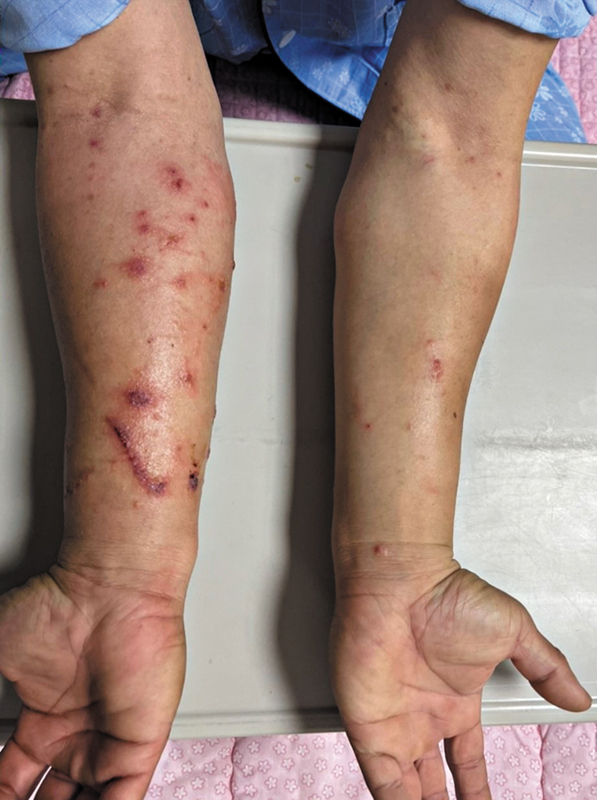
Clinical photograph of a 52-year-old male patient presenting with forearm wounds. The image shows a linear scratch wound on the right arm, surrounded by macules and blisters. A few macules and blisters are also visible on the left arm. These findings were observed upon the patient's admission to the hospital, 3 days after exposure to the mulberry tree.

Upon physical examination, the right arm, the site of the initial injury, exhibited pronounced swelling and a significant number of macules and blisters, whereas the left arm was almost unaffected, displaying minimal signs of macules and blisters ( Fig. 1 ). Upon admission, laboratory tests revealed a white blood cell count of 6.0 × 10 ^3^ /μL, hemoglobin level of 14.0 g/dL, and platelet count of 243 × 10 ^3^ /μL. Additionally, C-reactive protein (CRP) was 0.9 mg/dL, and the glycated hemoglobin (HbA1c) level was 7.3%. Considering the clinical presentation and suspecting cellulitis initially, we obtained a wound culture and commenced treatment with intravenous amoxicillin–clavulanate. The bullae were de-roofed and the wounds dressed with Bactigras (Smith & Nephew, United Kingdom) and a polyurethane foam. Antihistamines were prescribed for itching. Owing to the severity and nature of his symptoms, the patient was hospitalized for continued observation and treatment.

**Fig. 2:**
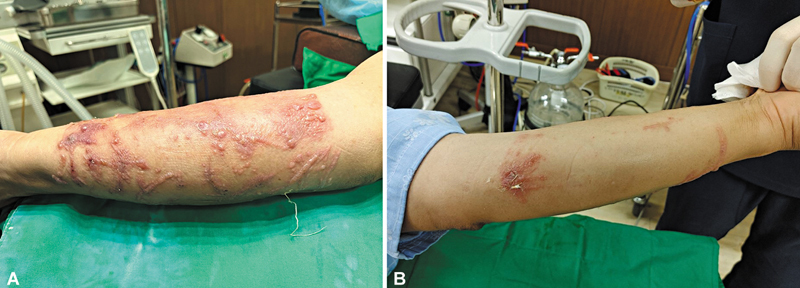
Clinical photograph taken before biopsy procedure. Despite conservative treatment, the lesion on the right arm worsened, forming an erythematous patch with bullae ( **A** ), and the lesion on the left arm also increased in size ( **B** ). These findings were observed on the fourth day after exposure to the mulberry tree. The lesions were confined to the forearms, correlating with the patient's report of wearing short sleeves and gloves while working outdoors, indicating exposure to sunlight and foliage in these areas. A biopsy was conducted on the bullae located on the right arm.

During hospitalization, the lesions on his right forearm showed no signs of improvement, and new blisters accompanied by a rash developed on his left forearm, confined to areas not covered by clothing or gloves ( Fig. 2 ). In light of the lack of progress, an excisional biopsy was performed on the right arm. Further detailed history taking revealed that the patient had been working near a mulberry tree by a utility pole.

Based on the patient's clinical presentation and history, we made a provisional diagnosis of PPD prior to confirmation by biopsy. Consequently, we introduced oral steroids into his treatment regimen (methylprednisolone 4 mg twice daily) and applied topical steroid cream (0.3% prednisolone) to the wounds.

**Fig. 3:**
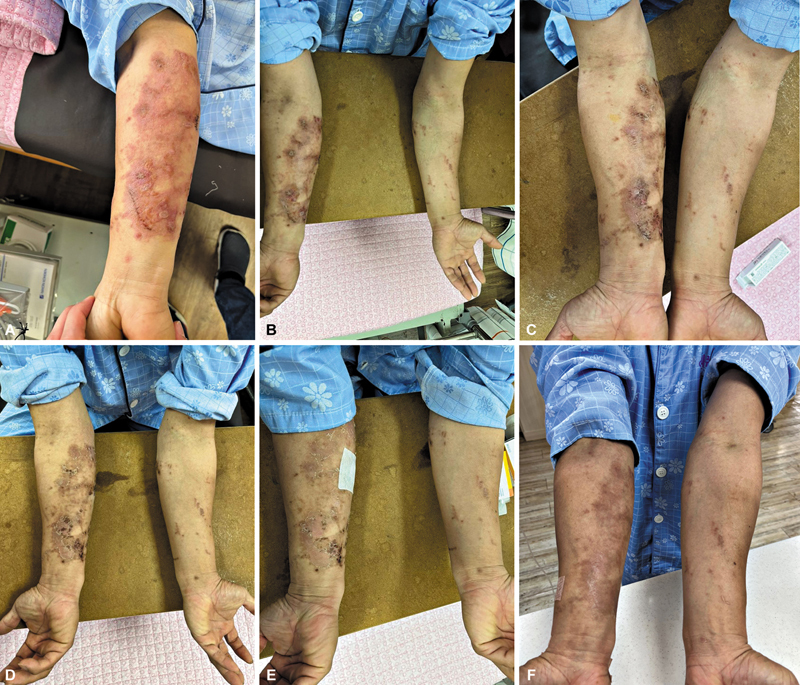
Serial follow-up clinical photographs documenting the treatment process. Day 1 after starting treatment for suspected phytophotodermatitis with oral and topical steroids, focusing on the right arm ( **A** ). Day 2 ( **B** ) demonstrates ongoing improvement. From Day 4 ( **C** ), dressings were minimized except for the biopsy site, which was covered with a foam dressing. The image from Day 7 ( **D** ) shows further healing of the lesions. On Day 10 ( **E** ), sutures at the biopsy site were removed. The final photograph on Day 16 ( **F** ) displays complete resolution of the lesions, leaving only hypopigmentation.

**Fig. 4:**
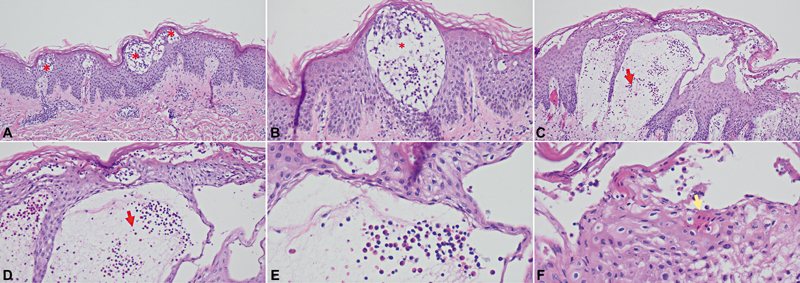
Histologic findings of the biopsy specimen. Intraepithelial blisters are identified ( **A, B** : red asterisk), along with areas showing subepithelial blisters ( **C, D** : red arrows). Within the subepithelial blister lesions, eosinophils and lymphocytes are observed ( **E** ), as well as apoptotic squamous cells in the peripheral areas of the subepithelial blisters ( **F** , yellow arrow). The magnifications are as follows: ( **A, C** ) at ×100, ( **B, D** ) at ×200, and ( **E, F** ) at ×400.

The patient exhibited noticeable improvement in clinical symptoms following the initiation of oral steroids and topical steroid cream, and all lesions resolved, leaving only hypopigmentation ( Fig. 3 ). The clinical diagnosis of PPD was later corroborated by the biopsy findings, which revealed intraepidermal and subepidermal blisters with mixed lymphocyte and eosinophil infiltration, acanthosis, and spongiosis ( Fig. 4 ). This outcome confirms the effectiveness of our clinical judgment and treatment approach. Written informed consent was obtained from the patient for the publication of this case report and any accompanying images.

## Discussion

**Fig. 5:**
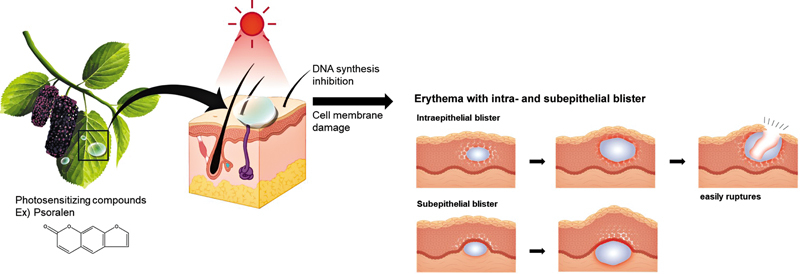
Schematic illustration depicting the pathophysiology of phytophotodermatitis. Photosensitizing compounds such as psoralens or furocoumarins, found in certain plants, become activated upon exposure to ultraviolet A radiation (wavelength 320–400 nm), leading to direct DNA damage and oxidative stress. This results in keratinocyte necrosis, intraepithelial and subepithelial blister formation, and inflammatory infiltration consisting of lymphocytes and eosinophils. The subsequent tissue injury manifests clinically as erythema, blistering, and characteristic skin lesions.

PPD, first described in 1942, is a phototoxic reaction occurring after contact with certain plants containing photosensitizing compounds (psoralens or furocoumarins) followed by exposure to UVA (wavelength 320–400 nm 6 7 9 ; Fig. 5 ). Unlike photoallergic reactions, PPD results directly from phototoxicity rather than immune mechanisms. Common plants causing PPD include species from the Apiaceae, Moraceae, and Rutaceae families, such as giant hogweed, mulberry, and lime ( Table 1 ).

**Table 1:** Common plants known to cause phytophotodermatitis

Family	Species	Common names
Apiaceae	*Ammi majus*	Bishop's weed
	*Apium graveolens*	Celery
	*Heracleum maximum*	Cow parsnip
	*Heracleum mantegazzianum*	Giant hogweed
	*Pastinaca sativa*	Parsnip
Moraceae	*Ficus carica*	Fig
	*Morus alba*	White mulberry
	*Morus rubra*	Red mulberry
	*Morus nigra*	Black mulberry
Rutaceae	*Citrus bergamia*	Bergamot orange
	*Citrus maxima*	Pomelo
	*Citrus latifolia*	Persian lime
	*Citrus aurantiifolia*	Mexican lime

Clinically, PPD presents initially as erythema in sun-exposed areas (face, neck, hands), typically appearing 24 to 48 hours after exposure, progressing to vesiculobullous lesions. 5 10 The rash frequently shows a linear pattern correlating with plant contact. Severe exposure may cause extensive blistering, peaking around 72 hours postexposure. 2 In our case, bullous lesions were initially observed only on the right arm at presentation (3 days postexposure). However, by the following day, the lesions on the right arm had worsened, and similar lesions had developed on the left arm. This asymmetric distribution may be attributed to differences in the degree of skin contact with the causative plant, potentially leading to variable furocoumarin absorption and subsequent lesion severity between the arms.

Histopathology typically demonstrates keratinocyte necrosis along with intraepidermal and subepidermal blister formation, accompanied by mixed inflammatory infiltrate 4 ( Fig. 5 ). The underlying pathophysiology involves direct DNA damage and oxidative stress due to activated psoralens interacting with UVA radiation, leading to cell injury and inflammation. 4

Diagnosis of PPD primarily relies on clinical presentation, highlighting the necessity of careful differential diagnosis from conditions such as cellulitis, allergic contact dermatitis, drug eruptions, or even child abuse in pediatric cases. 3 8 A thorough patient history, especially regarding occupational and recreational activities involving plant exposure, is crucial for accurate identification and avoiding misdiagnosis 8 11 ( Table 2 ).

**Table 2:** Review of cases of phytophotodermatitis reported in the literature

Study (year)	Country, number of cases	Age/Sex	Detailed history	Wound description	Treatment	Exposed material
Pérez-Camelo et al (2022) 7	Colombia, 1	37/F	The patient applied rue ( *Ruta graveolens* ), followed by a regular shower and a tanning bed session.	Superficial second-degree burns on the neck, thorax, back, and upper and lower limbs, involving 85% of the TBSA a	ICU admission, serial debridement	Rue ( *Ruta graveolens* )
Redgrave and Solomon (2021) 2	United Kingdom, 1	46/M	Arborist pruning a common fig tree under intense sunlight on a hot day	Painful, circumferential, patchy erythema with blistering, involving approximately 8% of TBSA	De-roofed blisters, wound dressing, admission, IV amoxicillin–clavulanate	Fig ( *Ficus carica* )
Abugroun et al (2019) 10	United States, 1	26/M	Performing outdoor activities under direct sunlight, primarily involving the squeezing of limes	A non-pruritic erythematous skin eruption developed on the dorsum of both hands, progressing to large bullae over the middle and ring fingers.	Blister drained, oral antibiotics (tetracycline)	Lime (Rutaceae family)
Son et al (2017) 11	Republic of Korea, 5	57/F	Applied fig leaf decoction for psoriasis, then walked outside on a sunny day	Erythematous swollen patches with bullae on hands and feet	Systemic and topical steroids for 4 months	Fig
		69/F	Applied fig leaf remedy for onychomycosis, then sun exposure	Erythematous patches with bullae on photo-exposed areas of both feet	Systemic steroids, antihistamines, and topical antibiotics; resolved in 2 months	
		66/M	Soaked feet in fig leaf decoction for onychomycosis, then sun exposure	Well-demarcated erythematous swollen patches with bullae on both feet	Topical steroids; follow-up unavailable	
		87/M	Soaked feet in fig leaf decoction for onychomycosis, then outdoor exposure	Erythema, edema, vesicles, and bullae confined to both feet	Systemic and topical steroids	
		70/F	Soaked feet in fig leaf decoction day, then sun exposure	Erythema, edema, and large bullae on the foot dorsa	Systemic steroids, topical antibiotics for 7 days	
Baker et al (2016) 9	United Kingdom, 3	27/F	Contact with giant hogweed while horse riding and dog walking in short sleeves	Superficial dermal burn on left forearm, with focal deep dermal areas, involving 1% TBSA	Irrigation, debridement	Giant hogweed ( *Heracleum mantegazzianum* )
		11/M	Erythema (right cheek) and blistering (left forearm) after weed cutting on the river bank	Superficial dermal burn (left forearm), involving 0.5% TBSA	Topical steroids (betamethasone 0.1%) and wound dressing for 1 week	
		13/M	Contact with hogweed sap while visiting a nature reserve	Blistering erythema on hands, forearms, thighs, legs; 2% TBSA skin loss	Antibiotics (flucloxacillin and penicillin V), Topical steroid (clobetasone 0.05%), and emollient twice daily for 14 days (fragile areas)	
Chan et al (2010) 6	Ireland, 1	10/M	Full-thickness burn (right pretibia) 2 weeks after hogweed exposure during football	Blisters progressed to 15 × 6 cm eschar with erythema in 2 weeks	Oral antibiotics (clindamycin), wound dressing, topical steroids (eumovate), debridement, and split-thickness graft	Giant hogweed
Furniss and Adams (2007) 3	United Kingdom, 1	2/M	Contact with rue while playing in the garden	Erythema on the face and hands progressed to blistering with clear discharge; fever, vomiting, poor intake, and hand stiffness led to admission	De-roofed blisters, topical wound dressing for 10 days	Rue
Derraik and Rademaker (2007) 8	New Zealand, 2	?/M b	Two male arborists were exposed to fig tree sap while clearing branches in short sleeves on a sunny day	Swelling, erythema, and tenderness developed, followed by bullae on the forearm, wrist, and hand	Mild case: Managed with lavender oil and aloe vera; symptoms persisted >2 weeks but resolved without medical treatment Severe case: Treated with topical corticosteroids and oral NSAIDs; gradual improvement over 4 weeks	Fig
Wynn and Bell (2005) 1	United Kingdom, 2	28/M	Two male grounds workers developed an arm rash after roadside grass cutting on a hot, sunny day	Symmetrical vesicular rash on flexor arms; spared areas covered by sleeves and gloves	Self-limited	Hogweed
		44/M				

a Total body surface area.

b Age not specified in the report.

Management of PPD is usually symptomatic and self-limiting. 2 Mild cases typically respond well to analgesics and supportive care. Topical corticosteroids may be indicated for moderate reactions, while extensive lesions may necessitate systemic corticosteroids. Antibiotics should be administered if secondary bacterial infection is suspected. In this case, oral methylprednisolone was administered at 4 mg twice daily for the first 3 days. As the symptoms showed a favorable clinical response, the dose was tapered to 4 mg once daily in the morning starting on day 4 ( Fig. 3C ). Both oral and topical steroids were discontinued on day 7 ( Fig. 3D ).

This case emphasizes the clinical importance of meticulous history-taking and heightened awareness among health care professionals, especially in wound care settings. Initially misdiagnosed as cellulitis due to erythema and skin abrasion, the correct identification of PPD significantly influenced the patient's treatment and outcomes. Timely recognition and proper management can minimize complications, including persistent hyperpigmentation, underscoring the importance of preventive education for patients at risk.

## References

[1] Wynn P Bell S Phytophotodermatitis in grounds operatives Occup Med (Lond) 2005 55 05 393 395 16040770 10.1093/occmed/kqi053

[2] Redgrave N Solomon J Severe phytophotodermatitis from fig sap: a little known phenomenon BMJ Case Rep 2021 14 01 e238745 10.1136/bcr-2020-238745 PMC7813380 33462031

[3] Furniss D Adams T Herb of grace: an unusual cause of phytophotodermatitis mimicking burn injury J Burn Care Res 2007 28 05 767 769 17667834 10.1097/BCR.0B013E318148CB82

[4] Grosu Dumitrescu C Jîjie A-R Manea H C New insights concerning phytophotodermatitis induced by phototoxic plants Life (Basel) 2024 14 08 1019 39202761 10.3390/life14081019 PMC11355232

[5] Safran T Kanevsky J Ferland-Caron G Mereniuk A Perreault I Lee J Blistering phytophotodermatitis of the hands after contact with lime juice Contact Dermatitis 2017 77 01 53 54 28612441 10.1111/cod.12728

[6] Chan J C Sullivan P J O'Sullivan M J Eadie P A Full thickness burn caused by exposure to giant hogweed: delayed presentation, histological features and surgical management J Plast Reconstr Aesthet Surg 2011 64 01 128 130 20399165 10.1016/j.bjps.2010.03.030

[7] Pérez-Camelo J S Barrios V Gómez-Ortega V Tanning beds, rue, and major burns: An alarming association Plast Reconstr Surg Glob Open 2022 10 02 e4106 35169530 10.1097/GOX.0000000000004106 PMC8830838

[8] Derraik J G Rademaker M Phytophotodermatitis caused by contact with a fig tree ( *Ficus carica* ) N Z Med J 2007 120 1261 U2720 17867224

[9] Baker B G Bedford J Kanitkar S Keeping pace with the media; Giant Hogweed burns - A case series and comprehensive review Burns 2017 43 05 933 938 28041748 10.1016/j.burns.2016.10.018

[10] Abugroun A Gaznabi S Natarajan A Daoud H Lime-induced phytophotodermatitis Oxf Med Case Rep 2019 2019 11 470 472 10.1093/omcr/omz113 PMC6902619 31844529

[11] Son J H Jin H You H S Five cases of phytophotodermatitis caused by fig leaves and relevant literature review Ann Dermatol 2017 29 01 86 90 28223753 10.5021/ad.2017.29.1.86 PMC5318534

